# Zerumbone Attenuates Lipopolysaccharide‐Induced Acute Lung Injury by Suppressing the NLRP3/Caspase‐1/GSDMD Signalling Pathway

**DOI:** 10.1111/jcmm.70873

**Published:** 2025-10-06

**Authors:** Yun‐Jie Xu, Fei‐Fei Fang, Guo‐Qiang Zhao, Wei‐Yan Yu, Hong‐Yan Han, Hong Teng, Jun‐Ning Lyu, Jian‐Feng Wang

**Affiliations:** ^1^ Zhejiang Chinese Medical University Hangzhou China; ^2^ Department of Respiratory Medicine Shaoxing Second Hospital Shaoxing China; ^3^ College of Medicine Shaoxing Vocational & Technology College Shaoxing China; ^4^ Department of Obstetrics and Gynecology Shaoxing Maternal and Child Health Hospital Shaoxing China; ^5^ Department of Applied Statistics Sydney Smart Technology College, Northeastern University at Qinhuangdao Qinhuangdao China; ^6^ Department of Respiratory Diseases The First Affiliated Hospital of Zhejiang Chinese Medical University (Zhejiang Provincial Hospital of Chinese Medicine) Hangzhou China

**Keywords:** acute lung injury, caspase‐1, GSDMD, lipopolysaccharide, NLRP3, pyroptosis, zerumbone

## Abstract

A cyclic sesquiterpene with 11 carbon atoms is the primary component of essential oils from the wild ginger species, *Zingiber zerumbet* . It exhibits pharmacological effects, including anti‐inflammatory and antioxidant properties. However, limited information exists regarding its role in pyroptosis during acute lung injury (ALI). Herein, we investigated how zingerone affects pyroptosis in a murine lipopolysaccharide (LPS)‐induced ALI model established via intraperitoneal zerumbone (Zer) administration. Bronchoalveolar lavage fluid, serum, and lung tissue samples were collected after 24 h. Haematoxylin and eosin staining was performed to assess lung tissue damage. Western blot analysis and real‐time quantitative polymerase chain reaction (qRT‐PCR) were used to quantify protein expression levels associated with pyroptosis. Before LPS exposure, mouse alveolar epithelial (MLE)‐12 cells were pretreated with 10 μM Zer for 2 h and then incubated with 1 ng/mL LPS for an additional 24 h. Zer treatment reduced lung tissue injury, inflammation, and oxidative stress in model mice. Zer counteracted pyroptosis in the alveolar epithelial cells of these mice. In MLE‐12 cells, Zer significantly inhibited oxidative stress and inflammation. Zer suppressed NLRP3/Caspase‐1/GSDMD signalling, pivotal in pyroptosis, mitigating ALI progression. A potential novel therapeutic agent for ALI inhibited the NLRP3/GSDMD signalling pathway involved in pyroptosis.

## 1 Introduction

Acute lung injury (ALI) is a life‐threatening condition of the lungs characterised by elevated pulmonary epithelial permeability, inflammatory cell infiltration, pulmonary oedema, and widespread alveolar damage. This condition can progress to a severe respiratory ailment known as acute respiratory distress syndrome, characterised by high rates of illness and fatality [1, 2]. One of the most common triggers of ALI is a severe infection caused by specific molecular components known as lipopolysaccharides (LPS), which are found on the outer surface of gram‐negative bacterial cells. Although pharmacological interventions offer some therapeutic benefits to patients, they are often accompanied by significant side effects and fail to reduce mortality effectively [3]. Therefore, identifying potent therapeutic targets and devising highly effective treatments with low toxicity are crucial for mitigating the effects of ALI.

Alveolar epithelial cells are the primary cellular targets of damage in ALI. Sustained oxidative stress and inflammatory dysregulation are pivotal pathogenic mechanisms in LPS‐induced lung epithelial cell injury [4, 5]. In ALI cases triggered by LPS, oxidative stress is disrupted, as LPS stimulates the production of reactive oxygen species (ROS), particularly hydrogen peroxide and oxygen, facilitated by xanthine oxidase. Additionally, LPS enhances the formation of malondialdehyde (MDA), a notable derivative of ROS [6]. Moreover, LPS, a vital component of the outer layer of gram‐negative bacterial cells, can induce lung damage and trigger intense inflammatory reactions [7]. Remarkably, 5α‐androst‐3β,5α,6β‐Triol exhibits anti‐inflammatory and antioxidant properties in lung epithelial and endothelial cells exposed to LPS by suppressing the activation of the NF‐κB and p38 mitogen‐activated protein kinase (MAPK) signalling cascades, consequently reinforcing the structural integrity of the lung epithelial barrier [8]. Stellate ganglion blocks alleviate LPS‐induced ALI by suppressing oxidative stress and reducing lung inflammation through the regulation of the SIRT3 signalling cascade [9]. Resolvin D1 exerts anti‐inflammatory effects by inhibiting aberrant neutrophil recruitment, stimulating neutrophil apoptosis, and alleviating oxidative stress‐induced injury through upregulation of heme oxygenase‐1 (HO‐1) expression [10]. Thus, exploring effective therapeutic strategies to mitigate oxidative stress and inflammation is crucial for treating LPS‐induced ALI.

Prolonged oxidative stress and inflammatory responses create a vicious cycle that triggers a specific type of programmed cell death known as pyroptosis, characterised by cellular enlargement, vacuole development, and eventual membrane disruption. Pyroptosis is initiated by inflammasome activation, notably the proteolysis of gasdermin D (GSDMD), leading to the development of membrane permeability pores [11]. The NLRP3 inflammasome, a member of the nucleotide‐binding oligomerisation domain‐like receptor family with a pyrin domain, triggers the self‐cleavage of pro‐caspase‐1, thereby activating caspase‐1. Upon activation, caspase‐1 catalyses the cleavage of GSDMD, yielding its N‐terminal portion. This fragment triggers the formation of membrane pores, leading to the secretion of inflammatory mediators, membrane disruption, and pyroptosis [12, 13]. The peptide LL‐37, the mouse ortholog of cathelicidin‐related antimicrobial peptide (CRAMP), protects against LPS‐induced lung damage. This is accomplished by suppressing the expression of constituents within the canonical NLRP3/Caspase‐1/GSDMD signalling cascade and its subsequent inflammatory mediators in alveolar epithelial cells [14]. Notably, deficiency of the basic helix–loop–helix family member e40 suppresses GSDMD‐mediated pyroptosis and mitigates ALI [15]. Additionally, inhibiting sphingosine kinase 1 alleviates LPS‐induced ALI by impeding endothelial cell pyroptosis [16]. Therefore, identifying potent therapeutic approaches to alleviate oxidative stress, inflammation, and pyroptosis is crucial for the management of LPS‐induced ALI.

Zerumbone (Zer), a naturally occurring phytochemical originally isolated in 1960 from the essential oil of the traditional medicinal plant ginger, possesses a broad spectrum of pharmacological properties and is frequently used in herbal medicine [17, 18]. Zer exhibits significant anticancer, anti‐inflammatory, and antioxidant activities that contribute to its widespread therapeutic applications [19, 20, 21]. Despite its beneficial properties, the influence of Zer on oxidative stress, inflammatory responses, and pyroptosis induction in alveolar epithelial cells stimulated by LPS has yet to be investigated. Consequently, this study aimed to elucidate the function of Zer in suppressing oxidative stress, inflammatory processes, and the NLRP3/Caspase‐1/GSDMD signalling cascade leading to pyroptosis in alveolar epithelial cells exposed to LPS.

## 2 Materials and Methods

### 2.1 Lipopolysaccharide‐Induced Mouse Model

Male C57BL/6 mice (all animal procedures were approved by the Animal Care and Use Committee of Shaoxing Hospital under Project Number SYXK2023‐0089) were randomly assigned to six groups: a phosphate‐buffered saline (PBS) + vehicle control, PBS + Zer (30 mg/kg) group [22], LPS + vehicle, LPS + Zer (20 mg/kg), LPS + Zer (30 mg/kg), and LPS + Zer (40 mg/kg). The mice received intratracheal injections of LPS (5 mg/kg) for 1 h, followed by intraperitoneal injections of either vehicle or Zer. Twenty‐four hours after LPS treatment, the mice were euthanised, and serum, bronchoalveolar lavage fluid (BALF), and lung tissue samples were collected for comprehensive analyses. Ethical approval for all animal experiments was granted by the Animal Care and Use Committee of Shaoxing Hospital (Approval Number: SYXK2023‐0089).

### 2.2 Histopathological Analysis

Pulmonary tissue samples were immobilised in a 4% paraformaldehyde solution and subsequently encapsulated in paraffin wax to produce thin slices ranging from 3 to 5 μm in thickness. The sections were stained with eosin and haematoxylin, and then dehydrated using a series of increasingly concentrated ethanol solutions. After clearing with xylene, the slices were mounted onto slides using a neutral balsamic mounting medium. Pathological changes in lung tissue were subsequently observed under a light microscope (Leica, Wetzlar, Germany).

### 2.3 Bronchoalveolar Lavage Fluid Collection and Analysis

The respiratory tissue of the right lung of each animal underwent three lavage procedures using 0.6 mL of PBS solution sourced from Sigma‐Aldrich (St. Louis, Michigan, USA). The collected BALF was centrifuged at 3000 rpm for 5 min using a Thermo Fisher Scientific centrifuge (Waltham, MA, USA). The supernatant was then evaluated for protein concentration, and the cellular precipitate was enumerated.

### 2.4 MLE‐12 Cells

The mouse alveolar epithelial cells MLE‐12 (ATCC, Manassas, VA, USA) were cultured in Dulbecco's Modified Eagle Medium (Gibco, Grand Island, NY, USA) supplemented with 10% foetal bovine serum (Gibco) and 1% penicillin/streptomycin (Gibco) at 37°C and 5% CO_2_ atmosphere. The final concentrations of the drugs used for cell treatment were as follows: Zer (10 μM) [23] and nigericin (20 μM) [24].

### 2.5 Analysis of Biochemical Indexes

The biochemical markers lactate dehydrogenase (LDH), MDA, and superoxide dismutase (SOD) were assayed according to the manufacturer's guidelines (Jiancheng Bioengineering Institute, Nanjing, China).

### 2.6 Real‐Time Quantitative PCR (RT‐qPCR) Analysis

Total RNA was isolated using TRIzol reagent. Subsequently, 1 μg of RNA was converted into cDNA through reverse transcription employing a transcription reversal kit sourced from Thermo Fisher Scientific. Quantitative polymerase chain reaction (PCR) analysis was executed with SYBR Green‐based PCR Master Mix on a Sigma‐Aldrich‐manufactured LightCycler 480 II real‐time PCR platform. The relative abundances of target genes were quantified by applying the 2^−ΔΔCt^ calculation technique, with GAPDH as the internal reference gene for normalisation. The primer sequences are shown in Table S1.

### 2.7 Determination of Cell ROS Levels

MLE‐12 cells were incubated with a 10 μM solution of the DCFH‐DA fluorescent indicator (Sigma‐Aldrich) at 37°C for 10 h. Subsequently, these cells were rinsed three times using PBS, and fluorescent images were acquired using an inverted fluorescent microscope (Zeiss, Oberkochen, Germany).

### 2.8 Cellular Immunofluorescence

Cells were immobilised using a 4% paraformaldehyde solution at room temperature for 15 min, followed by permeabilisation with 0.1% Triton X‐100 solution at room temperature for 20 min. After three rinses with PBS, the cellular samples were blocked using a 5% goat serum solution at room temperature for 30 min. The specimens were incubated with the primary antibody in a refrigerated environment at 4°C overnight. The next day, the cells were labelled with the corresponding fluorescent secondary antibodies at room temperature for 2 h, followed by three additional rinses with PBS. Finally, the slides were mounted using a fluorescent mounting medium containing 4′,6‐diamidino‐2‐phenylindole. Images were captured using a fully automated upright fluorescence microscope (Zeiss).

### 2.9 Western Blot

Total proteins from lung tissue and MLE‐12 cells were extracted using radioimmunoprecipitation assay buffer. The proteins were separated by sodium dodecyl sulfate‐polyacrylamide gel electrophoresis and transferred onto a PVDF membrane. The membrane was sealed with a 5% skim milk powder solution for 2 h. Subsequently, it was incubated overnight with the specific primary antibody required for the experiment. On the second day, the membrane was incubated with the corresponding secondary antibody, and immunoblots were detected using an advanced chemiluminescence reagent (Elabscience) and visualised using imaging techniques. Antibodies used in this article are detailed in Table S2.

### 2.10 Statistical Analysis

GraphPad software (GraphPad Software Inc., San Diego, CA, USA) was utilised for graphing and statistical analysis. Measurement data were presented as mean ± standard deviation from three independent experiments. A one‐way analysis of variance was used to compare multiple groups, followed by Tukey's post hoc analysis.

## 3 Results

### 3.1 Elevated Levels of Oxidative Stress, Inflammatory Responses, and Pyroptotic Activity Were Noted in a Mouse Model of ALI Induced by LPS

**FIGURE 1:**
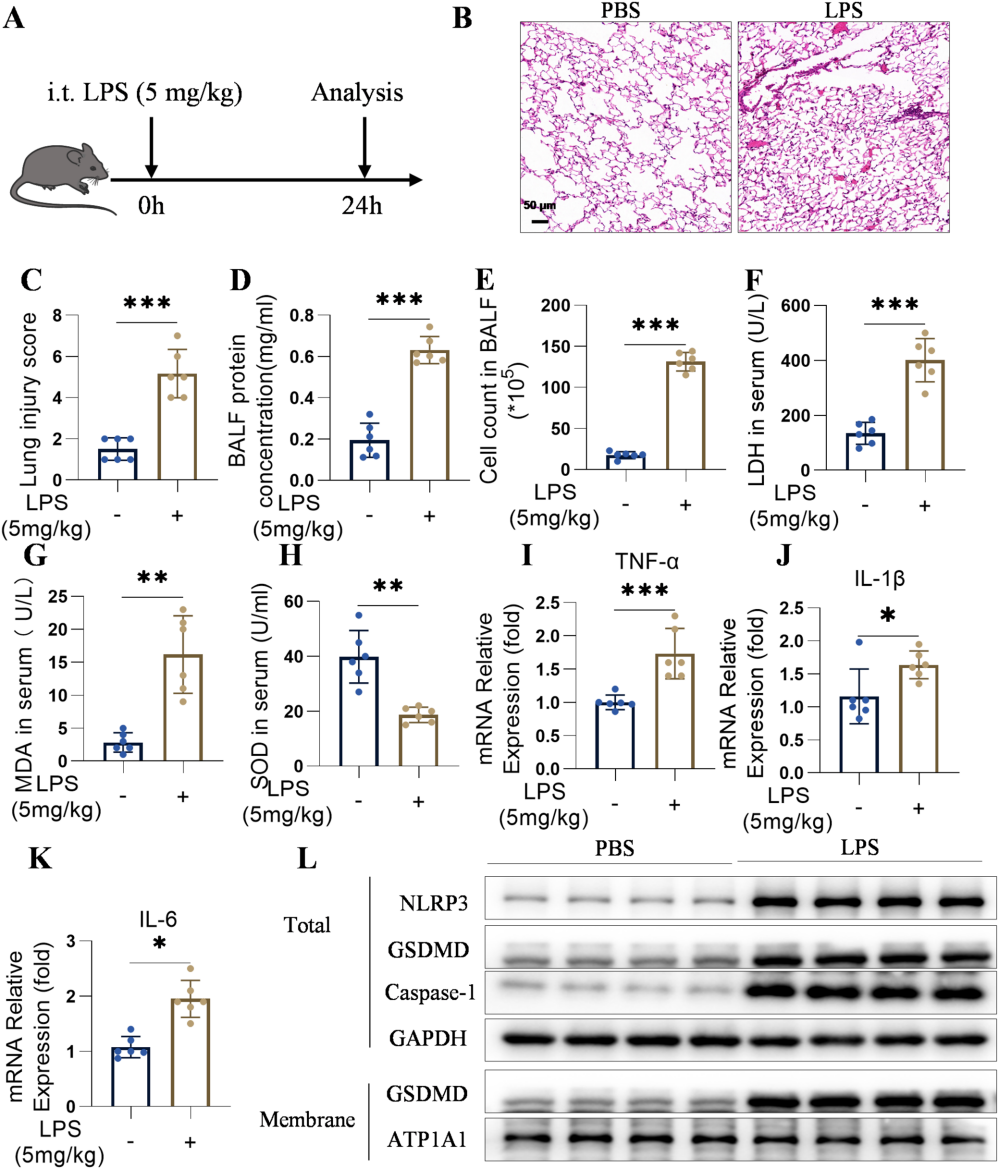
Elevated levels of oxidative stress, inflammatory responses, and pyroptotic activity were noted in a mouse model of ALI induced by LPS. (A) Establishment of the LPS‐induced ALI model. (B, C) Representative images of H&E staining lung sections (scale bar [50 μm]) (*n* = 6). (D, E) Total cell number and protein concentration in BALF (*n* = 6). (F–H) LDH, MDA, and SOD levels in mouse serum were measured using biochemical index kits (*n* = 6). (I–K) mRNA IL‐1β, TNF‐α, and IL‐6 expressions in lung tissue (*n* = 6). (L) Levels of the NLRP3 Caspase‐1, GSDMD, ATP1A1, and GAPDH proteins in the lung tissue (*n* = 4). The results were displayed as the mean ± standard error of the means and analysed using one‐way ANOVA followed by Tukey's multiple comparison test for further analysis. *p < 0.05, **p < 0.01, and ***p < 0.001.

To investigate the relationship between oxidative stress, inflammation, pyroptosis, and ALI, a mouse model of ALI was established through intratracheal instillation of LPS (Figure 1A). Control mice were administered PBS. LPS administration resulted in augmented inflammatory infiltration within the lung tissue compared to that in the PBS‐treated controls (Figure 1B,C). Notably, protein concentrations and cellularity in BALF were markedly elevated in LPS‐induced ALI mice compared to controls (Figure 1D,E). To quantify oxidative stress in ALI, levels of LDH, MDA, and SOD activity were measured. LPS treatment significantly increased LDH and MDA levels while reducing SOD activity (Figure 1F,H), indicating increased oxidative stress. Since oxidative stress can precipitate inflammatory responses, levels of TNF‐α, IL‐1β, and IL‐6 were measured and found to be upregulated in LPS‐induced lung tissues (Figure 1I–K). Additionally, the impact of pyroptotic processes on LPS‐induced ALI was examined. A significant elevation in the abundance of the NLRP3 inflammasome, Caspase‐1 protease, and GSDMD proteins in the lungs of mice subjected to LPS treatment, in contrast to the control group (Figure 1L for visual representation). These findings underscore the importance of elucidating the roles and regulatory pathways of oxidative stress, inflammatory responses, and pyroptotic mechanisms in LPS‐induced ALI.

### 3.2 Exposure to LPS Led to Enhanced Oxidative Stress, Inflammatory Responses, and Pyroptosis in MLE‐12 Cells

**FIGURE 2:**
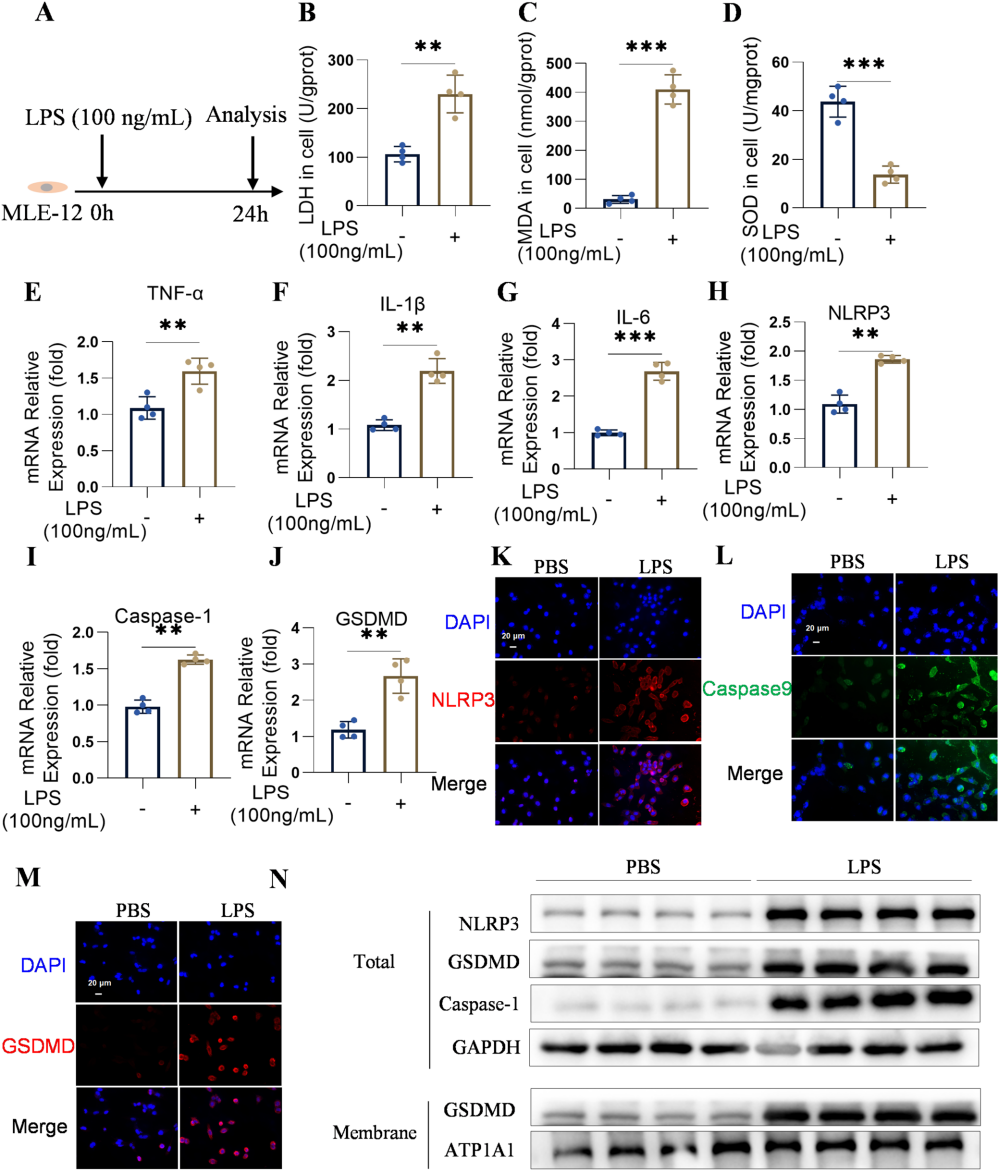
Exposure to LPS led to enhanced oxidative stress, inflammatory responses, and pyroptosis in MLE‐12 cells. (A) LPS stimulation in MLE‐12 cells. (B–D) LDH, MDA, and SOD levels in MLE‐12 cells (*n* = 4). (E–J) mRNA levels of IL‐1β, TNF‐α, IL‐6, NLRP3, Caspase‐1, and GSDMD expression in MLE‐12 cells (*n* = 4). (K–M) Immunofluorescence images of NLRP3, Caspase‐1, and GSDMD in MLE‐12 cells, scale bar = 20 μm (*n* = 4). (N) Levels of NLRP3, Caspase‐1, GSDMD, ATP1A1, and GAPDH proteins in lung tissue (*n* = 4). The results were presented as the mean ± standard error of the mean and analysed through one‐way ANOVA followed by Tukey's multiple comparison tests for further analysis, **p < 0.01, and ***p < 0.001.

In lung tissue, the alveolar epithelium is the predominant cell type. We investigated the effect of LPS on alveolar epithelial cells, specifically MLE‐12 cells (Figure 2A). LPS exposure led to elevated LDH and MDA levels, accompanied by a reduction in SOD activity in MLE‐12 cells (Figure 2B–D). Using qPCR, we analysed the mRNA expression of TNF‐α, IL‐1β, and IL‐6 and found that LPS induced the production of these inflammatory cytokines (Figure 2E–G). Subsequently, we assessed the extent of pyroptosis in LPS‐treated MLE‐12 cells. The mRNA results demonstrated increased levels of NLRP3, Caspase‐1, and GSDMD in LPS‐stimulated MLE‐12 cells (Figure 2H–J). These findings were corroborated by western blotting and immunofluorescence analyses, which indicated LPS‐induced pyroptosis in MLE‐12 cells (Figure 2K–N).

### 3.3 ZER Exhibits Inhibitory Effects on Inflammation, Oxidative Stress, and Pyroptotic Cell Death in a Mouse Model of ALI Induced by LPS

**FIGURE 3:**
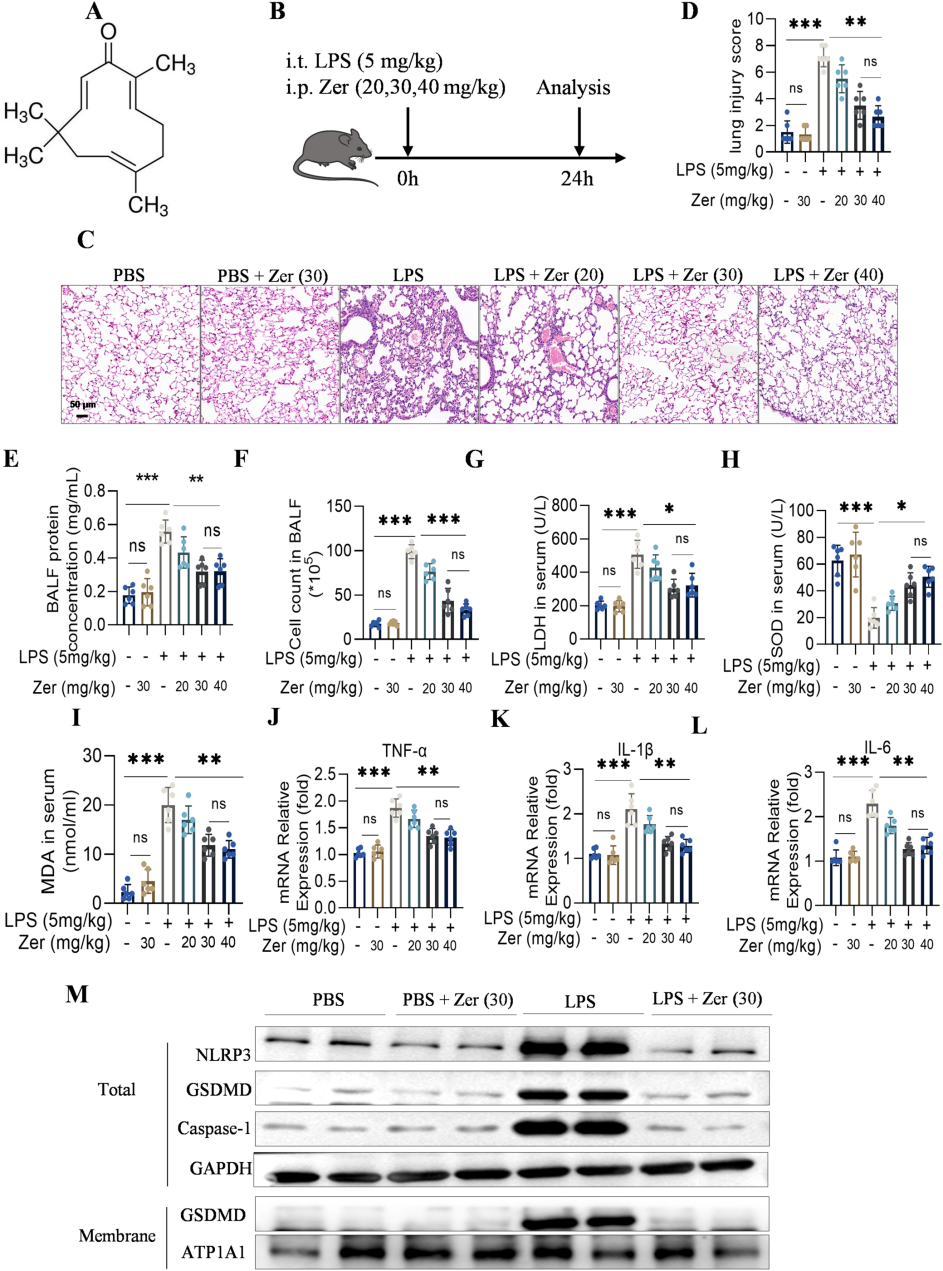
Zer exhibits inhibitory effects on inflammation, oxidative stress, and pyroptosis in a mouse model of ALI induced by LPS. (A) Zer structural formula. (B) Establishment of LPS‐induced ALI and Zer treatment groups. (C, D) Representative images of H&E staining lung sections (scale bar [50 μm]) (*n* = 6). (E, F) Total cell number and protein concentration in BALF (*n* = 6). (G–I) LDH, MDA, and SOD levels in mice serum (*n* = 6). (J–L) mRNA expression of IL‐1β, TNF‐α, and IL‐6 in lung tissue (*n* = 6). (M) Levels of NLRP3, Caspase‐1, GSDMD, ATP1A1, and GAPDH proteins in the lung tissue (*n* = 4). The results were displayed as the mean ± standard error of the mean and analysed using one‐way ANOVA followed by Tukey's multiple comparison test for further analysis. ns, not significant; *p < 0.05, **p < 0.01, and ***p < 0.001.

LPS‐induced mice were administered Zer to evaluate its role in ALI (Figure 3A,B). We found that Zer significantly decreased lung injury, protein concentration, and cell counts in BALF in a dose‐dependent manner (Figure 3C–F). Additionally, Zer administration reduced LDH and MDA levels and increased SOD activity in LPS‐treated mice (Figure 3G–I), suggesting that Zer modulates oxidative stress in ALI. Moreover, Zer inhibited the LPS‐induced increases in mRNA levels of TNF‐α, IL‐1β, and IL‐6 in mice (Figure 3J–L). Zer significantly reduced LPS‐induced expression of pyroptosis‐related proteins (Figure 3M), demonstrating that Zer regulates inflammation, oxidative stress, and pyroptosis in LPS‐induced ALI.

### 3.4 Zer Exhibits Inhibitory Properties Against Inflammation and Oxidative Stress in MLE‐12 Cells When Exposed to LPS

**FIGURE 4:**
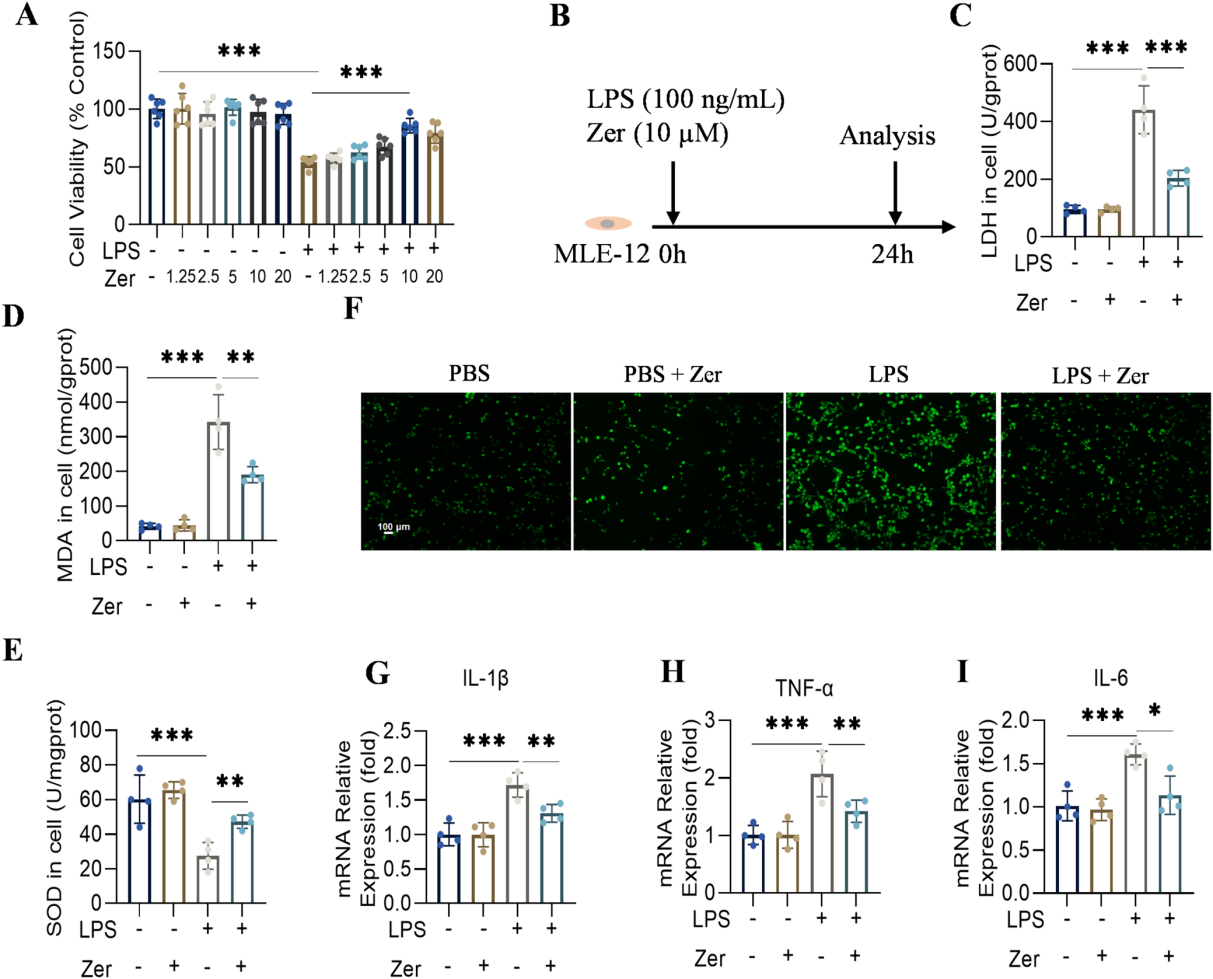
Zer exhibits inhibitory properties against inflammation and oxidative stress in MLE‐12 cells exposed to LPS. (A) CCK8 assay was performed to detect the effect of Zer on the viability of MLE‐12 cells induced by LPS (*n* = 6). (B) LPS stimulation and Zer pretreatment scheme for MLE‐12 cells. (C–E) LDH, MDA, and SOD levels in MLE‐12 cells (*n* = 4). (F) Cytosolic ROS generation in MLE‐12 cells, scale bar = 100 μm (*n* = 4). (G–I) The mRNA levels of IL‐1β, TNF‐α, and IL‐6 in MLE‐12 cells (*n* = 4). The results are presented as the mean ± standard error of the mean and evaluated using a one‐way ANOVA followed by Tukey's multiple comparison test for further analysis. *p < 0.05, **p < 0.01, and ***p < 0.001.

We treated LPS‐stimulated MLE‐12 cells with Zer to validate its effects in vitro (Figure 4A–B). Zer administration reduced LDH and MDA levels and increased SOD activity in LPS‐treated MLE‐12 cells (Figure 4C–E), suggesting that Zer modulates oxidative stress. Pretreatment with Zer reduced the ROS levels in MLE‐12 cells (Figure 4F). Zer administration suppressed increases in TNF‐α, IL‐1β, and IL‐6 levels in LPS‐induced MLE‐12 cells (Figure 4G–I), consistent with the ALI mouse model results.

### 3.5 Zerumbone Suppresses Pyroptosis in LPS‐Induced MLE‐12 Cells

**FIGURE 5:**
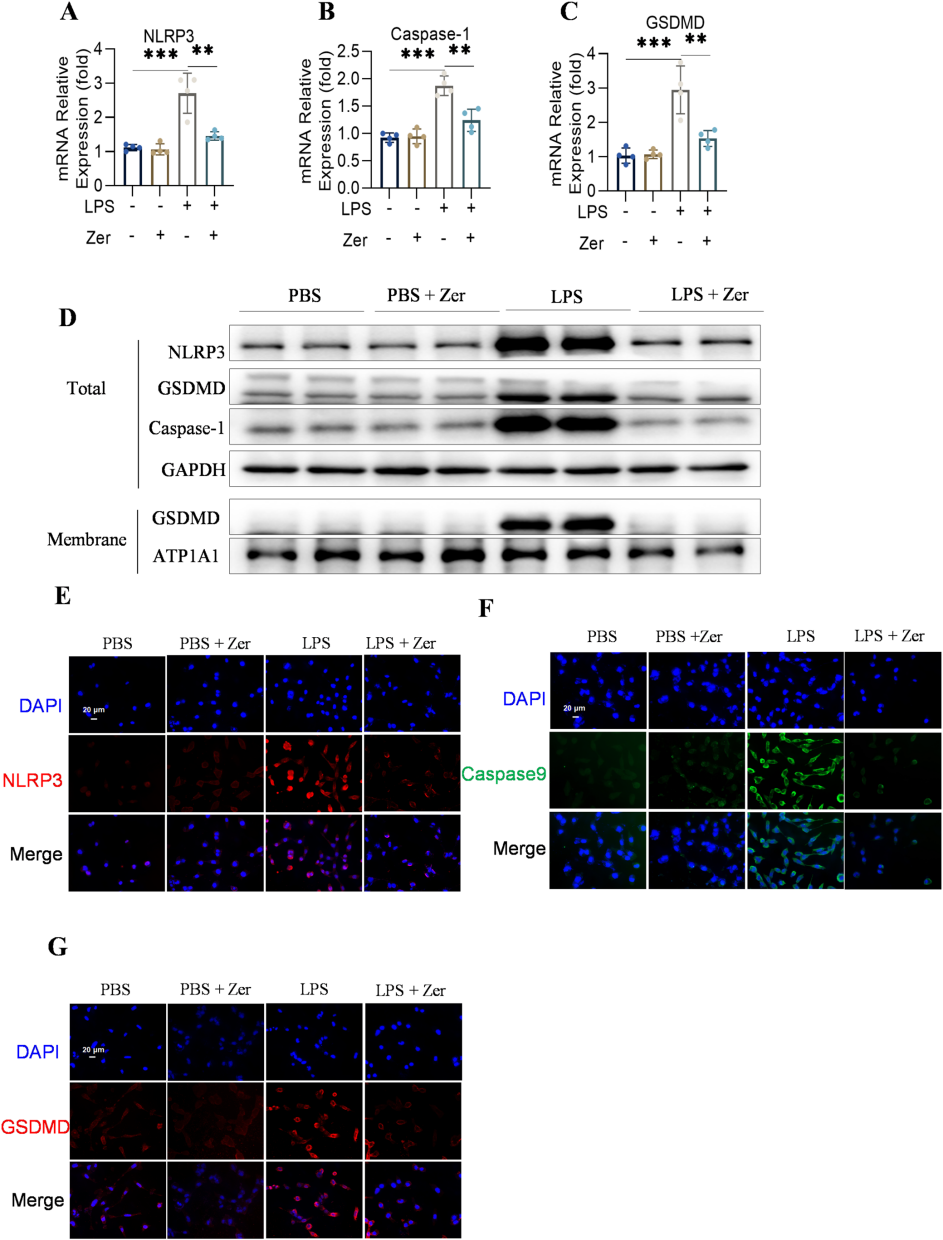
Zer suppressed pyroptosis in LPS‐induced MLE‐12 cells. (A–C) mRNA level of NLRP3, Caspase‐1, and GSDMD in MLE‐12 cells (*n* = 4). (D) Levels of NLRP3, Caspase‐1, GSDMD, ATP1A1, and GAPDH proteins in MLE‐12 cells (*n* = 4). (E–G) Representative immunofluorescence images of NLRP3, Caspase‐1, and GSDMD in MLE‐12 cells, scale bar = 20 μm (*n* = 4). The results are represented as the mean ± standard error of the mean and evaluated using one‐way ANOVA followed by Tukey's multiple comparison procedure for further analysis. **p < 0.01 and ***p < 0.001.

We performed in vitro experiments using MLE‐12 cells to investigate the role of Zer in pyroptosis. Zer administration reversed the enhanced pyroptosis in LPS‐induced MLE‐12 cells, including increases in NLRP3, Caspase‐1, and GSDMD expression (Figure 5A–C). Western blotting and immunofluorescence showed consistent results, indicating that Zer alleviated LPS‐induced pyroptosis in MLE‐12 cells (Figure 5D–G). These findings demonstrate that Zer plays a key role in the pathogenesis of ALI, which may be mediated by the regulation of pyroptosis.

### 3.6 Activation of NLRP3 Abolished the Beneficial Actions of Zer in Mitigating Oxidative Stress and Inflammatory Responses in MLE‐12 Cells

**FIGURE 6:**
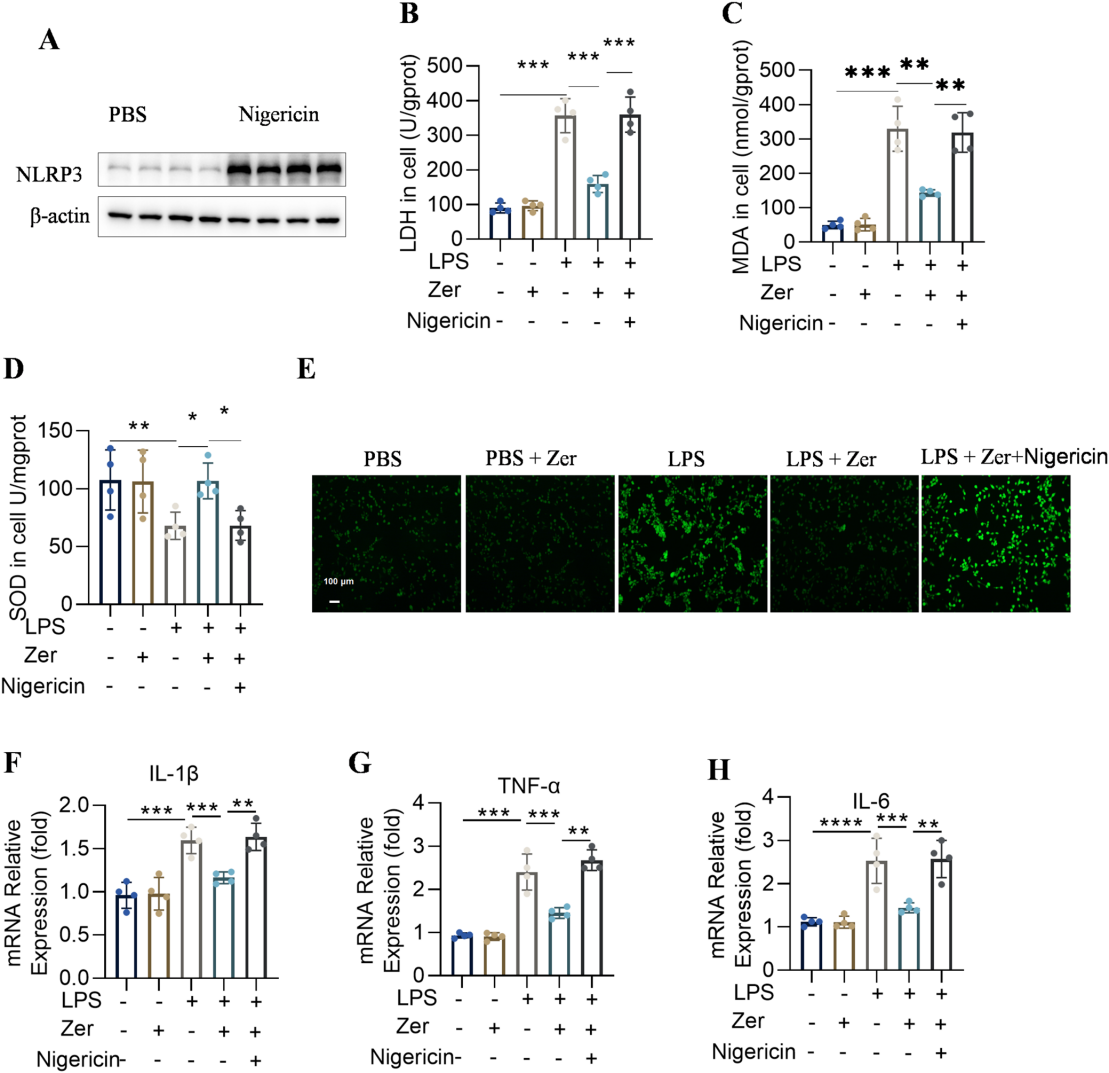
Activation of NLRP3 abolished the beneficial actions of Zer in mitigating oxidative stress and inflammatory responses in MLE‐12 cells. (A) Levels of NLRP3 and GAPDH proteins in MLE‐12 cells (*n* = 4). (B–D) LDH, MDA, and SOD levels in MLE‐12 cells (*n* = 4). (E) Cytosolic ROS generation in MLE‐12 cells, scale bar = 100 μm (*n* = 4). (F–H) The mRNA expression of IL‐1β, TNF‐α, and IL‐6 in MLE‐12 cells (*n* = 4). The results are displayed as the mean ± standard error of the mean and analysed using one‐way ANOVA followed by Tukey's multiple comparison test for further analysis. *p < 0.05, **p < 0.01, and ***p < 0.001.

Nigericin upregulated NLRP3 expression in MLE‐12 cells (Figure 6A). Zer did not restore the changes in oxidative damage (LDH, MDA, SOD, and ROS) after nigericin treatment (Figure 6B–E). Additionally, Zer administration could not reduce the increased TNF‐α, IL‐1β, and IL‐6 levels in LPS‐induced MLE‐12 cells when NLRP3 agonists were used (Figure 6F–H), demonstrating that NLRP3 activation eliminated the protective function of Zer in LPS‐induced MLE‐12 cells.

### 3.7 The Protective Influence of Zer on Pyroptotic Cell Death in MLE‐12 Cells Was Negated by NLRP3 Activation

**FIGURE 7:**
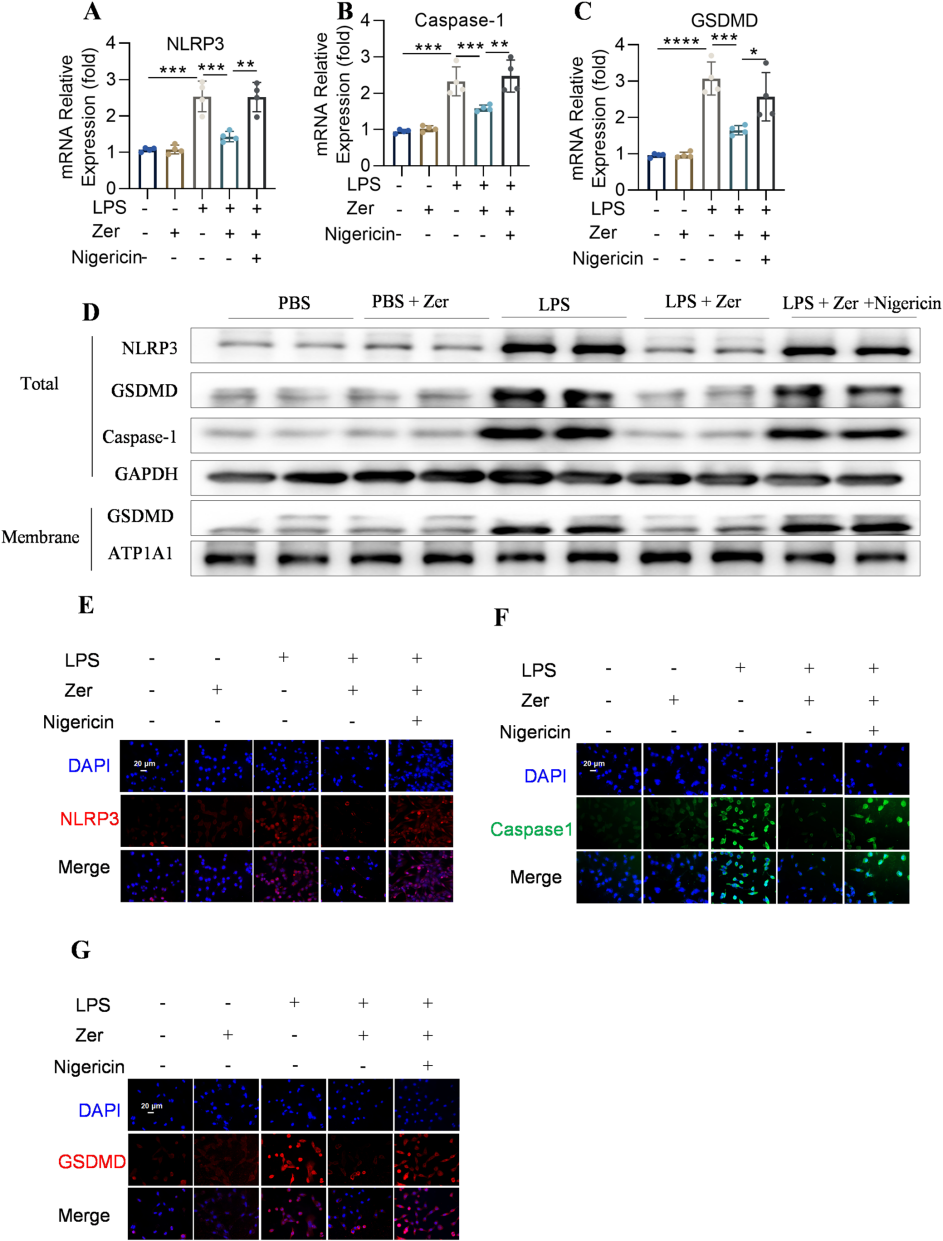
The protective influence of Zer on pyroptotic cell death in MLE‐12 cells was nullified by NLRP3 activation. (A–C) mRNA expression levels of NLRP3, Caspase‐1, and GSDMD in MLE‐12 cells (*n* = 4). (D) Levels of NLRP3, Caspase‐1, GSDMD, ATP1A1, and GAPDH proteins in MLE‐12 cells (*n* = 4). (E–G) Representative immunofluorescence images showing NLRP3, Caspase‐1, and GSDMD in MLE‐12 cells, scale bar = 20 μm (*n* = 4). The results are presented as the mean ± standard error of the mean and analysed using one‐way ANOVA followed by Tukey's multiple comparison test for further analysis. *p < 0.05, **p < 0.01, and ***p < 0.001.

The results demonstrated that pretreatment with nigericin diminished the decrease in the mRNA levels of NLRP3, Caspase‐1, and GSDMD (Figure 7A–C). Western blot and immunofluorescence analyses revealed that Zer reduced LPS‐induced pyroptosis in MLE‐12 cells, whereas the NLRP3 agonist diminished the effect of Zer (Figure 7D–G), demonstrating that Zer plays a key role in the pathogenesis of ALI and may mediate the regulation of pyroptosis (Figure S1). These observations demonstrate the critical role of Zer in the pathogenesis of ALI through the regulation of the NLRP3/Caspase‐1/GSDMD pyroptosis signalling pathway.

## 4 Discussion

ALI is characterised by the dysregulation of oxidative stress and aberrant inflammatory responses. LPS, an integral component of the outer membrane in Gram‐negative bacteria, serves as a potent pathogenic mediator capable of inducing pulmonary injury and exacerbating inflammatory cascades [7, 25]. Herein, we utilised an ALI model induced via LPS administration for subsequent experimental investigations, consistent with previous findings [26]. Zer, the first sesquiterpene isolated from ginger, found in regions including India, Malaysia, Indonesia, Bangladesh, Hawaii, China, and Thailand, shows promising antioxidant and anti‐inflammatory properties in prior studies. In this study, administration of Zer ameliorated LPS‐induced oxidative stress and inflammatory responses in pulmonary epithelial tissues by inhibiting the NLRP3/Caspase‐1/GSDMD signalling pathway, thereby attenuating apoptosis of pulmonary epithelial cells.

Sustained oxidative stress and inflammation are pivotal mechanisms underlying ALI [27]. The pathological mechanisms of ALI are complex and involve factors such as neutrophil proliferation, activation, and chemotaxis, which damage lung vascular endothelial and alveolar epithelial cells. This damage results in elevated levels of ROS and proteases and activates inflammatory signalling pathways, thereby enhancing lung microvascular permeability and epithelial cell injury [28, 29]. Therefore, mitigating oxidative stress and inflammation is crucial for ALI treatment. Telmisartan reduces LPS‐induced increases in MDA levels and decreases glutathione peroxidase levels, thereby alleviating oxidative stress in mice with LPS‐induced ALI [30]. LPS induces high ROS concentrations and disrupts the oxidative stress balance in the body, significantly reducing SOD and CAT activities while increasing MDA levels. Conversely, ST1926 ameliorates LPS‐induced oxidative stress imbalance, thereby improving ALI [31]. Our results are consistent with previous studies. Mechanistically, sulfasalazine exerts protective effects by inhibiting the NF‐κB inflammatory signalling pathway [32]. Pre‐administration of a respiratory immunostimulant notably decreases LPS‐triggered inflammatory indicators, including inflammatory agent generation, macrophage accumulation, NF‐κB activation in the lung parenchyma, and elevated inflammatory cell counts in BALF, alleviating bronchial and pulmonary inflammation [33]. Pretreatment with Zer inhibits the LPS‐induced p38 MAPK/JNK‐IκB/NF‐κB pathway, suppressing the expression of pro‐inflammatory cytokines, including IL‐1β and macrophage inflammatory protein (MIP)‐2, and alleviating ALI [34]. The outcomes of our research correspond with previous findings, showing that LPS stimulates the secretion of pro‐inflammatory cytokines TNF‐α, IL‐1β, and IL‐6 in lung tissue and MLE‐12. Administration of Zer attenuated the secretion of these cytokines and thereby mitigated LPS‐induced inflammatory cell infiltration in the lungs.

Sustained oxidative stress and inflammatory responses culminate in pyroptosis, a programmed cell death mechanism intricately linked to pro‐inflammatory signalling cascades. Pyroptotic cells undergo a sequence of events characterised by cellular swelling, lysis, chromatin condensation, and membrane pore formation, leading to the release of intracellular pro‐inflammatory cytokines [35]. The activation of pyroptosis through the inflammasome cascade is a prevalent phenomenon [36], with NLRP3 as its most prominent member. The NLRP3 complex comprises a specialised recognition receptor, the apoptosis‐associated speck‐like adaptor protein, and caspase‐1. Inflammatory responses and ROS are potent activators of the NLRP3 inflammasome [37, 38]. Therefore, inhibiting the expression of NLRP3 is the key to reducing pyroptosis. Both MCC950 and VX‐765 demonstrated significant therapeutic efficacy in attenuating hypoxia‐induced cardiomyocyte injury by suppressing NLRP3 inflammasome expression, thereby inhibiting pyroptosis and reducing myocardial oxidative stress and inflammation [39]. In septic mice with ALI, pharmacological inhibition of NLRP3 activity significantly attenuated LPS‐triggered pulmonary damage, alveolar oedema, and inflammatory cascades [40]. Consistent with these findings, a prior study demonstrated that LPS exposure elevates NLRP3 protein levels in both murine lungs and alveolar epithelial cells, subsequently inducing alveolar epithelial pyroptosis. In the present study, we observed that Zer administration significantly suppressed NLRP3 expression, attenuated LPS‐induced pulmonary epithelial apoptosis, and consequently mitigated oxidative stress and inflammatory responses in lung tissues. To further validate the inhibitory effect of Zer on NLRP3 protein expression, we employed the known NLRP3 activator nigericin to determine whether pharmacological activation of NLRP3 could abrogate the protective effects of Zer against LPS‐triggered lung injury. These results collectively suggest that Zer alleviates LPS‐induced oxidative stress and inflammation in ALI by inhibiting the NLRP3/caspase‐1/GSDMD axis implicated in pyroptotic signalling.

While acknowledging the limitations of this study, future research should prioritise three key areas to strengthen the translational relevance and mechanistic rigour: first, the pyroptosis effect of Zer on ALI lung epithelial cells was verified by using Caspase‐1 specific overexpression mice; second, evaluating the prophylactic potential of Zer by administering it intraperitoneally prior to LPS challenge to assess preventive efficacy; and third, developing NLRP3 inflammasome‐specific overexpression mouse models to elucidate the causal relationship between NLRP3 hyperactivation and disease progression in ALI.

In conclusion, our study revealed for the first time that Zer alleviates LPS‐induced oxidative stress and inflammation and ameliorates lung injury by inhibiting the NLRP3/Caspase‐1/GSDMD pyroptosis signalling pathway (Figure S1). These findings highlight the novel functional properties of Zer and suggest it as a promising therapeutic candidate for the treatment of LPS‐induced lung injury.

## Author Contributions

**Yun‐Jie Xu:** data curation (lead), formal analysis (lead), writing – original draft (lead). **Fei‐Fei Fang:** conceptualization (equal), funding acquisition (equal), investigation (equal), writing – review and editing (equal). **Guo‐Qiang Zhao:** methodology (supporting), project administration (supporting), software (supporting), writing – review and editing (supporting). **Wei‐Yan Yu:** resources (equal), supervision (equal), visualization (equal), writing – review and editing (supporting). **Hong‐Yan Han:** methodology (equal), resources (equal), writing – review and editing (equal). **Hong Teng:** methodology (equal), resources (equal), writing – review and editing (equal). **Jun‐Ning Lyu:** investigation (equal), project administration (equal), writing – review and editing (equal). **Jian‐Feng Wang:** data curation (lead), formal analysis (lead), investigation (equal), project administration (equal), writing – original draft (lead).

## Ethics Statement

All animal procedures were approved by the Animal Care and Use Committee of Shaoxing Hospital under Project Number SYXK2023‐0089.

## Consent

The authors have nothing to report.

## Conflicts of Interest

The authors declare no conflicts of interest.

## Supporting information


**Figure S1:** Schematic diagram of the protective mechanism of zerumbone against LPS‐induced ALI. This illustration depicts the proposed molecular mechanism by which zerumbone (yellow ellipse) attenuates LPS (purple burst)‐induced ALI. LPS triggers oxidative stress, inflammatory cytokine release, and pyroptosis (NLRP3/caspase‐1/GSDMD pathway), collectively contributing to ALI pathogenesis. Green arrows denote activation pathways, while black T‐bars indicate inhibitory effects of zerumbone on LPS‐mediated oxidative stress, inflammation, and pyroptosis.
**Table S1:** Primers for real‐time quantitative PCR analysis.
**Table S2:** List of primary antibodies used in Western blots.

## Data Availability

The data that support the findings of this study are available from the corresponding author upon reasonable request.
