# Supplementary material for: Zerumbone Attenuates Lipopolysaccharide‐Induced Acute Lung Injury by Suppressing the NLRP3/Caspase‐1/GSDMD Signalling Pathway
Source: J Cell Mol Med. 2025 Oct 6;29(19):e70873. doi: 10.1111/jcmm.70873 (PMC12500414; doi:10.1111/jcmm.70873)
Supplement: Supplementary file 1 — Figure S1: Schematic diagram of the protective mechanism of zerumbone against LPS‐induced ALI. This illustration depicts the proposed molecular mechanism by which zerumbone (yellow ellipse) attenuates LPS (purple burst)‐induced ALI. LPS triggers oxidative stress, inflammatory cytokine release, and pyroptosis (NLRP3/caspase‐1/GSDMD pathway), collectively contributing to ALI pathogenesis. Green arrows denote activation pathways, while black T‐bars indicate inhibitory effects of zerumbone on LPS‐mediated oxidative stress, inflammation, and pyroptosis. Table S1: Primers for real‐time quantitative PCR analysis. Table S2: List of primary antibodies used in Western blots. [file JCMM-29-e70873-s001.docx]

**SUPPORTING INFORMATION**

**Supplementary Table S1**. Primers for Real-Time Quantitative PCR Analysis

| Gene | Primer | Sequence |
| --- | --- | --- |
| *IL-1β* | Forward | 5‘-CAAGGAGAACCAAGCAACGA-3’ |
|  | Reverse | 5‘-TTTCATTACACAGGACAGGTATAGA-3’ |
| *IL-6* | Forward | 5‘-ACTTCCATCCAGTTGCCTTCTTGG-3’ |
|  | Reverse | 5‘-TTAAGCCTCCGATTGTGAAGTG-3’ |
| *TNFα* | Forward | 5‘-AGGTTCTCTTCAAGGGACAA-3’ |
|  | Reverse | 5‘-GACTTTCTCCTGGTATGAGATAG-3’ |
| *NLRP3* | Forward | 5‘-TCACAACTCGCCCAAGGAGGAA-3’ |
|  | Reverse | 5‘-AAGAGACCACGGCAGAAGCTAG-3’ |
| *Caspase-1* | Forward | 5‘-GGCACATTTCCAGGACTGACTG-3’ |
|  | Reverse | 5‘-GCAAGACGTGTACGAGTGGTTG-3’ |
| *GAPDH* | Forward | 5‘-TGTGATGGGTGTGAACCACGAGAA-3’ |
|  | Reverse | 5‘-GAGCCCTTCCACAATGCCAAAGTT-3’ |

**Supplementary Table S2**. List of primary antibodies used in Western blots

| Antibody | Dilution |
| --- | --- |
| GSDMD (Cell Signalling Technology, #96458) | 1:1000 |
| ATP1A1 (Proteintech, 14418-1-AP) | 1:1000 |
| NLRP3 (Cell Signalling Technology, 15101S) | 1:1000 |
| Caspase-1 (ABclonal, A0964) | 1:1000 |
| GAPDH (Proteintech, 60004-1-Ig ) | 1:5000 |


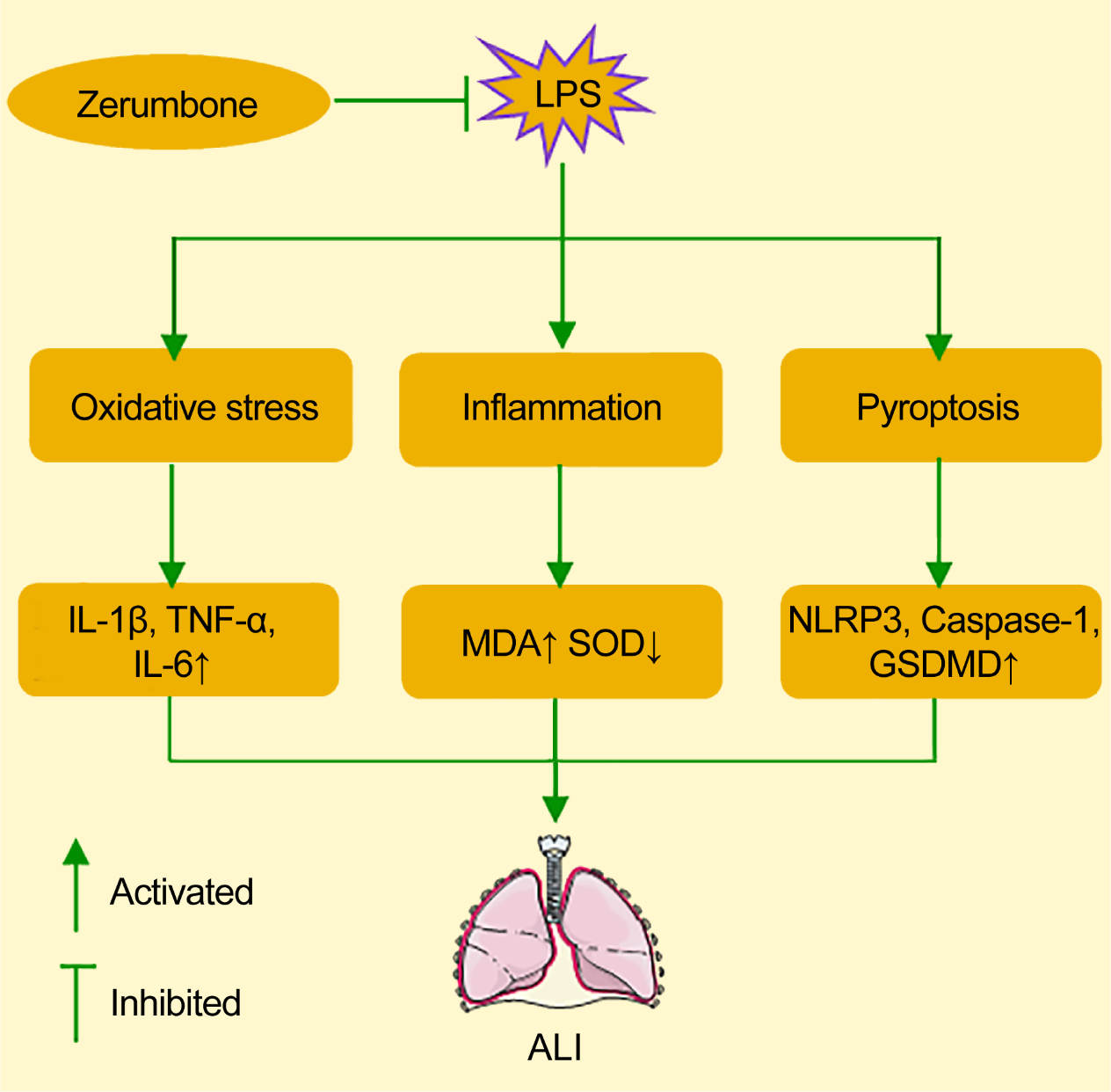


**Supplementary Figure 1**. Schematic diagram of the protective mechanism of Zerumbone against LPS-induced ALI. This illustration depicts the proposed molecular mechanism by which Zerumbone (yellow ellipse) attenuates LPS (purple burst)-induced ALI. LPS triggers oxidative stress, inflammatory cytokine release, and pyroptosis (NLRP3/caspase-1/GSDMD pathway), collectively contributing to ALI pathogenesis. Green arrows denote activation pathways, while black T-bars indicate inhibitory effects of Zerumbone on LPS-mediated oxidative stress, inflammation, and pyroptosis.
